# The Structure of Liquid and Amorphous Hafnia

**DOI:** 10.3390/ma10111290

**Published:** 2017-11-10

**Authors:** Leighanne C. Gallington, Yasaman Ghadar, Lawrie B. Skinner, J. K. Richard Weber, Sergey V. Ushakov, Alexandra Navrotsky, Alvaro Vazquez-Mayagoitia, Joerg C. Neuefeind, Marius Stan, John J. Low, Chris J. Benmore

**Affiliations:** 1 X-ray Science Division, Argonne National Laboratory, Argonne, IL 60439, USA; gallington@anl.gov (L.C.G.); lawrie.skinner@gmail.com (L.B.S.); rweber@matsdev.com (J.K.R.W.); 2 Argonne Leadership Computing Facility, Argonne National Laboratory, Argonne, IL 60439, USA; ghadar@anl.gov (Y.G.); vama@anl.gov (A.V.-M.); 3 Peter A. Rock Thermochemistry Laboratory and NEAT ORU, University of California at Davis, Davis, CA 95616, USA; svushakov@ucdavis.edu (S.V.U.); anavrotsky@ucdavis.edu (A.N.); 4 Chemical and Engineering Materials Division, Oak Ridge National Laboratory, Oak Ridge, TN 37830, USA; neuefeindjc@ornl.gov; 5 Global Security Sciences, Argonne National Laboratory, Argonne, IL 60439, USA; mstan@anl.gov; 6 Computing, Environment and Life Sciences, Argonne National Laboratory, Argonne, IL 60439, USA; jlow@anl.gov

**Keywords:** X-ray diffraction, neutron diffraction, molecular dynamics, liquid structure, amorphous materials, nanoparticles, hafnium oxide

## Abstract

Understanding the atomic structure of amorphous solids is important in predicting and tuning their macroscopic behavior. Here, we use a combination of high-energy X-ray diffraction, neutron diffraction, and molecular dynamics simulations to benchmark the atomic interactions in the high temperature stable liquid and low-density amorphous solid states of hafnia. The diffraction results reveal an average Hf–O coordination number of ~7 exists in both the liquid and amorphous nanoparticle forms studied. The measured pair distribution functions are compared to those generated from several simulation models in the literature. We have also performed ab initio and classical molecular dynamics simulations that show density has a strong effect on the polyhedral connectivity. The liquid shows a broad distribution of Hf–Hf interactions, while the formation of low-density amorphous nanoclusters can reproduce the sharp split peak in the Hf–Hf partial pair distribution function observed in experiment. The agglomeration of amorphous nanoparticles condensed from the gas phase is associated with the formation of both edge-sharing and corner-sharing HfO_6,7_ polyhedra resembling that observed in the monoclinic phase.

## 1. Introduction

Of all the high dielectric constant materials considered as possible replacements for SiO_2_ as a gate dielectric, the transition metal oxide HfO_2_ is the material of choice for resistive random-access memory (RAM) applications [1,2,3]. HfO_2_ can be grown as metastable amorphous phases on a silicon wafer; however, it is not a good glass former, and annealing it leads to crystallization [4]. While HfO_2_ offers good chemical stability, defects in the HfO_2_ crystalline bulk can be detrimental to the behavior of the devices, as they can lead to flat-band voltage instabilities [3]. A number of computer simulations have been performed in the literature to characterize and understand the atomic and electronic structure of amorphous HfO_2_ and the associated oxygen diffusion [1,2,3,4,5,6,7,8]. The majority of these simulations form amorphous or glassy phases by quenching from the melt, but there is a lack of experimental data available to benchmark the various approaches. In particular, several simulations have presented atom–atom pair distribution functions (PDFs) with significantly varying structures. Here, we present X-ray and neutron PDF data on both the melt and amorphous nanoparticles prepared by gas condensation that are directly comparable to these simulations. X-ray and neutron techniques are highly complementary in the case of metal oxides; while X-ray scattering is dominated by the cations, neutrons are sensitive to oxygen positions [9]. In this way, the combination of PDF results provide a rigorous test of structural models and highlight the strong influence of density on the atomistic structure and oxygen diffusion within amorphous HfO_2_ [1]. Along with changes in short-range order, medium range order, particularly connectivity between polyhedra, can change significantly with density. For amorphous HfO_2_, we show the distribution of corner to edge sharing HfO_n_ units has a large influence on the structural arrangements within the nanoparticles.

## 2. Materials and Methods

### 2.1. Sample Preparation

Spherical beads of monoclinic HfO_2_ were prepared by fusing HfO_2_ powder (Aldrich, St. Louis, MO, USA, 99.995% trace metal purity) into spheres using a 10.6 μm CO_2_ laser on a copper hearth. Amorphous HfO_2_ nanoparticle samples were prepared in 2010 and 2015 using a custom gas-phase condensation setup [10,11,12]. A CO_2_ laser beam operated at 40 W was focused on the surface of rotating HfO_2_ target (Materion, Mayfield Heights, OH, USA, 99.9% trace metal purity). Evaporation was performed under a controlled background O_2_ pressure (~0.1 Torr O_2_) to ensure formation of amorphous rather than crystalline material. Samples were recovered by transferring the synthesis chamber to an Ar-filled glove box and scraping the condensate from the substrate. The surface area of the samples was analyzed by nitrogen adsorption using the BET (Brunauer–Emmet–Teller) model. Detailed calorimetric measurements have been reported for anhydrous HfO_2_ samples prepared by this method and studied shortly after synthesis [12]. Two batches of amorphous HfO_2_ were used in this work: the first was an as-deposited sample with surface area 226 ± 1 m^2^/g (subsequently referred to as 2015); the second was a sample annealed after deposition at 300 °C with surface area 142 ± 1 m^2^/g (2010). These surface areas correspond to particle sizes of 2.8–3.5 and 4.4–5.5 nm, respectively, depending on the density values used for calculations. In the current study, samples were loaded into either polyimide (X-ray measurements) or quartz (neutron measurements) capillaries under ambient atmosphere.

### 2.2. High Energy X-ray Diffraction

X-ray total scattering data were collected on amorphous hafnia samples at beamline 6-ID-D at the Advanced Photon Source (Argonne National Laboratory) using an amorphous silicon area detector (PE-XRD1621, PerkinElmer, Waltham, MA, USA). Data were acquired using either 60.07 keV or 131.74 keV X-rays. The X-ray beam had a 0.5 × 0.5 mm cross-section. The 60 keV data were taken below the Hf K-edge at 65.351 keV at a nominal sample to detector distance of 50 cm (Q_max_ ~ 15 Å^−1^) to minimize fluorescence effects. The 131 keV data were collected at the same distance (Q_max_ ~ 26.5 Å^−1^), and a 3 mm brass plate was placed in front of the detector to reduce background from additional fluorescence above the K-edge. A renormalization factor of 15% was required to scale the 131 keV data set to agree with the 60 keV measurement. 

The precise sample to detector distance, beam center, detector tilt and rotation were determined by measuring the diffraction pattern of a crystalline standard CeO_2_ powder (NIST SRM 674b) and performing a calibration in *FIT2D* [13]. Reduction of two-dimensional images to one-dimensional scattering data was performed using *FIT2D* [13]. Faber–Ziman (atom–atom) pair distribution functions were extracted from total scattering data using *PDFgetX2* [14,15]. Corrections for background, Compton scattering, fluorescence, and oblique incidence were optimized in *PDFgetX2* [14,15]. Data were normalized to the average electron density given by the weighted sum of the independent atom X-ray form factors, thus yielding the X-ray structure factor S(Q) and total X-ray pair distribution function:

T(r) = 4 π*ρ*r∙G(r),

where *ρ* is the atomic number density and G(r) is the pair distribution function as defined by the Hannon–Howells–Soper formalism (Equation (30) in ref. [16]). In this manuscript, both G(r) and T(r) definitions are used: G(r) highlights the local structure and is readily comparable to simulation results, while T(r) emphasizes intermediate range ordering and allows for extraction of coordination numbers.

For the liquid state and variable temperature measurements, samples were levitated on either pure oxygen or pure argon gas and melted using a CO_2_ laser at beamline 6-ID-D at the Advanced Photon Source. Powder diffraction data were collected while cooling samples to room temperature in 100–200 K increments. Additional measurements were performed on molten samples at beamline 11-ID-C at the Advanced Photon Source. All levitated samples were illuminated with monochromatic 100.27 keV (0.123653 Å) X-rays. To minimize attenuation, the X-ray beam was centered on the top 0.5 mm of the ~2.5 mm diameter sample. To further reduce the effects of attenuation, only a 120° phi wedge centered vertically above the sample was analyzed. Data reduction and extraction of pair distribution functions were subsequently performed as described for the amorphous samples.

### 2.3. Neutron Diffraction

Amorphous hafnia samples were measured using neutron diffraction on the NOMAD beamline at the Spallation Neutron Source (Oak Ridge National Laboratory) [17]. Powder diffraction data were also collected on a solid ~2 mm diameter beads of pure HfO_2_ at room temperature. Standard corrections, including background subtraction and normalization to vanadium, were applied to the data using beamline software [18]. Neutron absorption resonances from Hf affected the data below a wavelength of 0.4 Å, resulting in issues for data in the region Q > 30 Å^−1^. Attempts to apply wavelength filters were unsuccessful, so all wavelengths were used and the data truncated at Q_max_ = 25.0 Å^−1^.

### 2.4. Ab Initio Molecular Dynamics Simulations

Ab initio molecular dynamics simulations were carried out with the Intel branch of the CP2K package as implemented in the QUICKSTEP module [19,20]. The exchange and correlation energies were estimated with the Perdew–Burke–Ernzerhof (PBE) functional [21]. Goedecker–Teter–Hutter PBE pseudopotentials for oxygen and hafnium were used, as distributed with CP2K [22]. The polarized double-zeta basis sets for hafnium and oxygen were taken from the MOLOPT basis set collection as distributed with CP2K [23]. A plane wave basis with a cutoff of 700 Rydbergs, a relative cutoff of 70 Rydbergs and 5 multigrids were used to fit the charge density. A time step of 1 fs was used to propagate the molecular dynamics trajectory. The constant number, pressure and temperature ensemble was used with a canonical sampling through velocity rescaling (CVSR) thermostat and barostat [24].

Simulations for the crystalline HfO_2_ polymorphs were initially run for 1 ps with a CVSR time constant of 10 fs to quickly approach the desired temperature and pressure. After equilibration, the trajectory was run for 10 ps with CVSR time constant of 1 ps after the temperature reached the desired temperature and pressure. The python library MDtraj was used to derive radial distribution functions of the crystalline and liquid forms from the trajectories [25].

### 2.5. Classical Molecular Dynamics Simulations

Classical molecular dynamics simulations of the cubic, tetragonal and monoclinic phases of HfO_2_ were carried out using LAMMPS (Sandia National Laboratory, Albuquerque, NM, USA; version 16 February 2016) [26]. The simulations were performed utilizing a recently developed LAMMPS implementation of the modified charge transfer potential model (CTIP) potential developed for multiple metal oxides [27,28,29]. Additional details regarding the CTIP potential are included in Appendix A. The simulation box for each phase was ~25 Å in each dimension and contained at least 1500 atoms (Table 1). Crystal structures obtained from ICSD database [30,31,32] were replicated 5 or 6 times along each axis. Periodic boundary conditions were applied for all 3 dimensions. Long range electrostatics were modeled using Ewald summation with a cut off of 12 Å and default convergence parameters [33]. The CTIP cut off was 20 Å. The Verlet integration method [34] was used with a time step of 0.5 fs. The charge relaxation procedure was used to minimize the electrostatic energy for every MD step. After the initial minimization, structures were equilibrated at temperatures between 300 K and 3000 K (100 K intervals). Utilizing NVT ensemble with Nose-Hover thermostat [35,36,37], equilibration runs of 100 ps were obtained. At each temperature, radial distribution functions for Hf–Hf, Hf–O and O–O were determined. Bond-angle distributions for Hf–O–Hf and O–Hf–O were also extracted.

The formation of a 3.2 nm diameter nanoparticle of amorphous HfO_2_ was simulated using LAMMPS with the forcefield and quenching procedure utilized in Broglia et al. [1]. In total, 356 Hf and 712 O atoms were randomly placed in a 3.2 nm sphere and the energy of the system was minimized. A molecular dynamics trajectory was run for 80 ps at 4000 K and quenched to 300 K in 740 ps (5 K/ps). The cluster was stabilized with 80 ns of molecular dynamics at 300 K. No restrictions were placed on the density of HfO_2_ in this simulation. A visualization of the molecular dynamics trajectory is provided in Video S1.

## 3. Results

### 3.1. X-ray and Neutron Diffraction of Amorphous HfO_2_

**Figure 1:**
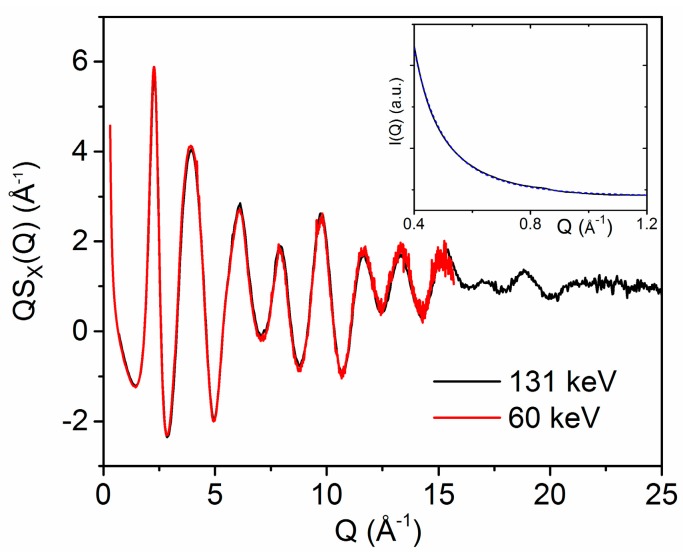
X-ray diffraction structure factors Q∙S_X_(Q) measured on the 2015 amorphous hafnia sample obtained using incident X-ray energies of 131 keV (black) and 60 keV (red). (Inset) Small angle scattering (Q = 0.4–1.2 Å^−1^) measured at 60 keV (blue) and fit corresponding to the equation y = a + b∙Q^−3.94^ (dashed line).

X-ray diffraction patterns of the as-deposited (2015) HfO_2_ sample reveal that the material is completely amorphous (Figure 1). Patterns of the sample annealed at 300 °C (2010) were almost identical, aside from a few weak Bragg peaks, indicating the presence of a small amount of crystalline material. The Q^−4^ rise in the small angle scattering region (Figure 1, inset) originates from a distribution of smooth particle interfaces, a few nm in size.

**Figure 2:**
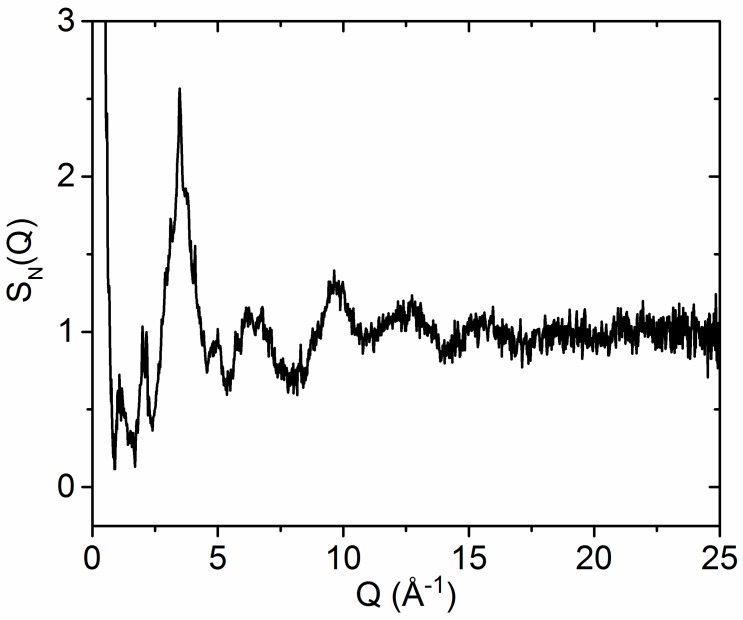
The neutron diffraction structure factor S_N_(Q) measured from the 2010 amorphous hafnia sample.

Neutron scattering from the 2010 sample (Figure 2) was significantly stronger than from the 2015 sample due to the larger amount of sample available. The neutron diffraction patterns were similar apart from a few Bragg peaks in the 2010 sample and some silica contamination from the capillary in the 2015 sample.

**Figure 3:**
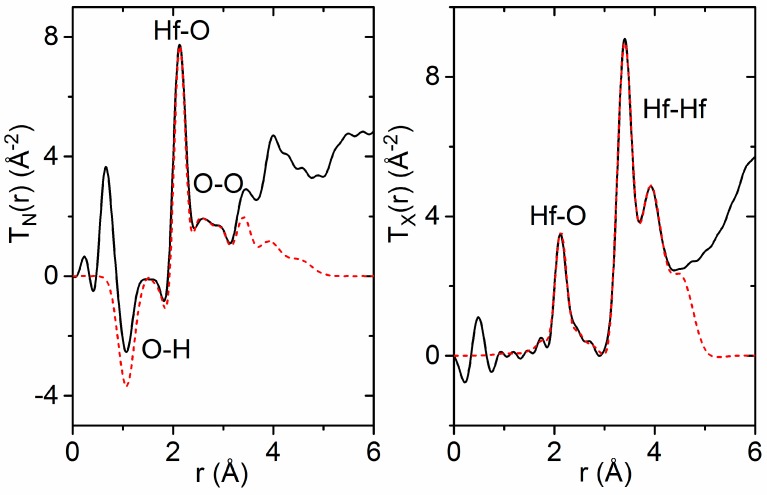
Simultaneous Gaussian fits (dashed red) to the measured total pair distribution functions (solid black), assuming a composition of (HfO_2_)_0.9_· (H_2_O)_0.1_. (**Left**) Total pair distribution function obtained from neutron data, T_N_(r). (**Right**) Total pair distribution function obtained from X-ray data, T_X_(r). The negative dips at 1.1 Å and 1.8 Å are assigned to intra-molecular hydrogen bonds in water and O–H groups within the amorphous hafnia network.

A comparison of the extracted T_N_(r) functions reveals two distinct dips at 1.07(1) Å and 1.81(1) Å in the amorphous hafnia sample that are not present in the pure hafnia monoclinic crystal structure (Figure 3). The negative direction of these features points towards the presence of hydrogen, as the bound coherent neutron scattering length for hydrogen is −3.74 fm [38], and the distances correspond to the intra-molecular and inter-molecular OH bond lengths in water [39]. However, if molecular H_2_O were present on the surface or in pores, an additional intra-molecular H–H peak would be present at ~1.5 Å in T_N_(r). Although this feature is not clearly observed in T_N_(r), a broad H–H peak may be hidden by peak overlap with O–H dips or convoluted with systematic errors at low r. Hydrogen may also be present in the form of O–H groups. 

A coordination number analysis was performed using NXFit [40]. The amount of assumed water in the sample was adjusted empirically until the best fit was obtained for both the neutron and X-ray data sets. This involved an iterative process, whereby the X-ray data were analyzed first assuming a composition (HfO_2_)_1−*x*_·(H_2_O)*_x_* to identify the location and magnitude of the Hf–O and Hf–Hf correlations, as X-rays are sensitive to heavier elements. Parameters describing these Hf correlations were held fixed while fitting to the neutron data, and the amount of H_2_O was varied. Finally, the fit was refined using both neutron and X-ray data simultaneously. The best fits were obtained when *x* < 0.1 [(HfO_2_)_0.9_· (H_2_O)_0.1_], which corresponds to a number density of 0.066(8) atoms Å^−3^. More dilute concentrations of water resulted in a 3% error in the Hf–O coordination number obtained from the X-ray fit.

### 3.2. X-ray Diffraction of Molten HfO_2_

**Figure 4:**
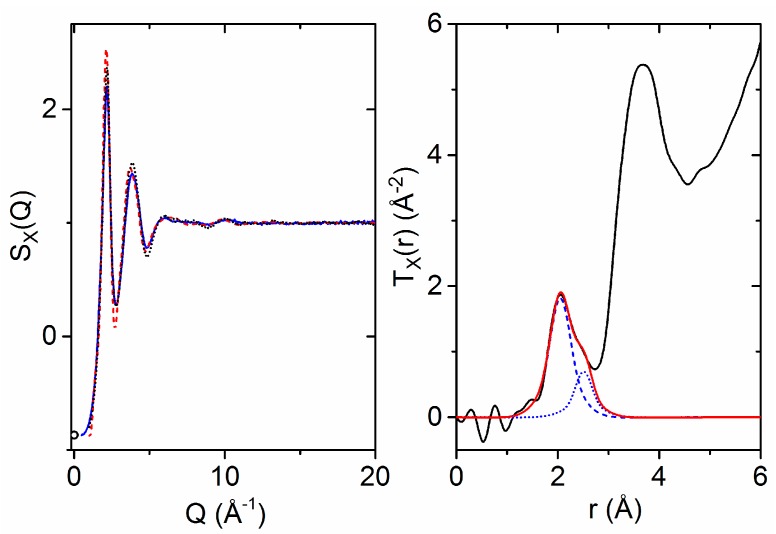
(**Left**) X-ray structure factors of molten HfO_2_ at 2900 °C measured on 11-ID-C (solid blue line) and at 3000 °C measured on 6-ID-D (black dots) compared to the results of the ab initio MD simulation (red dashes). The circle represents the isothermal compressibility limit at Q = 0 Å^−1^ [14]. (**Right**) X-ray weighted total pair distribution function (black) obtained via Fourier transform with a variable modification function applied [41,42] (Q_max_ = 20 Å^−1^) compared to individual Gaussian atom distributions (blue dashes/dots) convoluted with the X-ray form factors and their sum (red).

The structure factor for molten HfO_2_ at equilibrium is directly comparable to simulation. The X-ray structure factor of molten HfO_2_ at 2900(50) °C was measured out to a Q-value of 22.5 Å^−1^. Neutron diffraction data were not collected, as the melting point was too high to achieve given the long laser-to-sample distance, which extended over several meters. The first maximum in the X-ray structure factor is located at 2.16(2) Å^−1^, and only very minor structural oscillations are observed past 12 Å^−1^. The measured G_X_(r) has a first peak maximum at 2.05(1) Å, which is ascribed to Hf–O correlations. A distinct asymmetry is present in the first shell Hf–O peak (Figure 4). The second peak in T_X_(r) is broad, with a maximum at 3.67(2) Å, and is dominated by Hf–Hf correlations. Taking the Q = 0 X-ray weighting factors for simplicity, we note that the underlying O–O correlations have only a 3% weighting compared to the 67% weighting of the Hf–Hf correlations. The X-ray and neutron Faber–Ziman weighting factors for the partial pair distribution functions are:

S_XRAY_(Q = 0) = 67∙S_HfHf_(Q) + 30∙S_HfO_(Q) + 3∙S_OO_(Q), S_NEUTRON_(Q = 0) = 16∙S_HfHf_(Q) + 48∙S_HfO_(Q) + 36∙S_OO_(Q). (1)

From inspection of the low-Q and low-r limits, the number density is estimated to be 0.070(8) atoms Å^−3^. After considering the Q-dependent Hf–O weighting factor, two Gaussians were fit to the first peak in T_X_(r), yielding a Hf–O coordination number of 7.0(6). The large error can be attributed to both uncertainty in the density and overlapping correlations on the high-*r* side of the first shell. The two Gaussian peak fit shown in Figure 4 corresponds to Hf–O coordination numbers of 5.0 centered at 2.05 Å and 2.0 at 2.51 Å. Past 5 Å, the X-ray pair distribution function is characterized by an exponentially damped sinusoidal decay, with frequency corresponding to the 2.16 Å^−1^ first peak in S_X_(Q).

**Figure 5:**
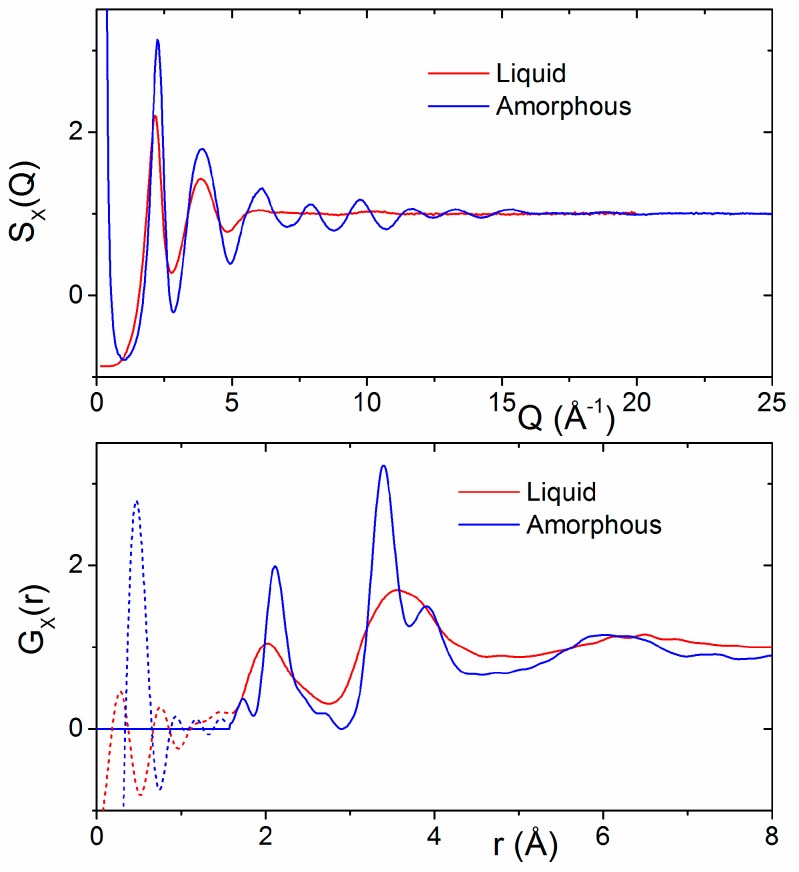
(**Top**) Comparison of the X-ray structure factors for liquid HfO_2_ and amorphous hafnia nanoparticles. (**Bottom**) A comparison of the corresponding X-ray pair distribution functions G_X_(r).

A comparison of the HfO_2_ melt X-ray structure factor and corresponding pair distribution function with those of amorphous hafnia is shown in Figure 5. Distinct oscillations in S(Q) for Q > 5 Å^−1^ appear in the amorphous signal that are not readily apparent in the liquid state. These correspond to increased ordering and are manifested in the T_X_(r) as a sharpening and lengthening of the Hf–O bond peak from 2.05(1) to 2.13(1) Å and the appearance of two well-defined peaks at 3.38(1) Å and 3.89(1) Å corresponding to Hf–Hf correlations. Oscillations were found to extend to at least 15 Å in real space for amorphous hafnia.

The coordination number (CN) analysis of the X-ray data for amorphous (HfO_2_)_0.9_·(H_2_O)_0.1_ sample using NXFit yields a Hf–O coordination number of 6.8(3) using three Gaussians (comprising of CN = 5.0 at 2.12 Å, 0.9 at 2.29 Å, and 0.9 at 2.87 Å).

## 4. Discussion

### 4.1. The Hf–O Phase Diagram

**Figure 6:**
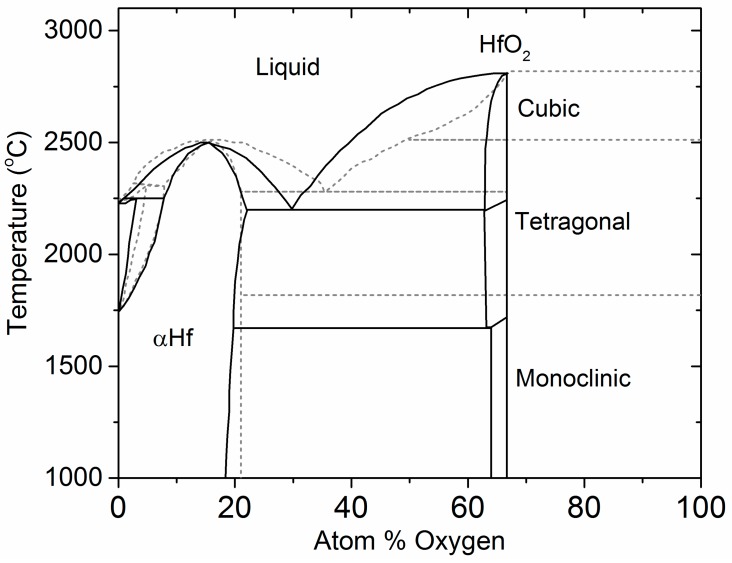
Assessment of the Hf–O phase diagram (solid line) [46] and DFT-based CALPHAD calculation of the Hf–O diagram (dashed line) [47].

The phase diagram of the Hf–O system has been studied extensively as part of an effort to understand thermal stability in refractory materials [43,44,45]. A phase diagram was proposed in 1990 based on thermodynamic data available at the time [46]. The phase diagram is complex, involving large solubility domains, eutectic and peritectic points, and allotropic phases of HfO_2_ (Figure 6). In addition, the Hf–O binary was assessed as part of thermal equilibria studies of ternary systems such as Hf–Si–O and Zr–Si–O. A more recently proposed phase diagram incorporates density functional theory (DFT) calculations and thermodynamic CALculation of PHAse Diagrams (CALPHAD) in addition to experimental information [47,48]. Further studies, also based on DFT methods, established the relative stabilities between monoclinic, tetragonal, and cubic phases of HfO_2_ with respect to cation dopants or oxygen vacancies.

### 4.2. Previous Experimental Studies on HfO_2_

The allotropic phases of HfO_2_ (monoclinic, tetragonal, and cubic) and associated transformations have been studied and the results summarized by Wang et al. [49]. At ambient temperature, HfO_2_ exists in a monoclinic crystal structure. It was demonstrated that the tetragonal-monoclinic athermal phase transformation occurs martensitically with a temperature hysteresis loop near 2023 K for pure HfO_2_ [50] and the hysteresis loop extending for 25 K for HfO_2_ [51]. Subsequent heating results in formation of the cubic phase at ~2973 K just prior to melting around 3073 K [31,45,52,53,54,55]. Wang et al. showed that the allotropic transformation temperatures are affected by particle size, impurities, stress, and the thermal history of materials [56].

In the monoclinic phase, Hf atoms are surrounded by seven oxygen atoms, with an asymmetric arrangement of Hf–O bond distances in the range 2.03 Å to 2.25 Å, corresponding to a mixture of seven-edge shared polyhedra and four-corner shared polyhedra in the unit cell [57]. In cubic HfO_2_, the Hf coordination increases to eight oxygen atoms at a bond distance of ~2.21 Å, and all the polyhedra are edge shared [31]. Additionally, in monoclinic HfO_2_, 50% of the O atoms have a coordination number equal to three and 50% have a coordination number equal to four. We find essentially the same numbers are observed both in the melt (O–Hf CN = 3.5(3)) and the amorphous solid (O–Hf CN = 3.4(3)); likewise, the distribution of bond lengths is highly asymmetric, with ~30% of the atoms involved in extended O–Hf bonds.

Experimental studies of the local structure of amorphous HfO_2_ are scant. X-ray diffraction and EXAFS studies on as-deposited sputtered thin films showed a rise in amplitude of the Hf–O and Hf–Hf correlations upon annealing [58,59]. X-ray pair distribution function studies on ZrO_2_, which is isostructural to HfO_2_, revealed that the short-range order of the amorphous phase resembles that of cubic ZrO_2_ rather than that of the tetragonal or monoclinic phases, with Zr–O and Zr–Zr coordination numbers of 6.7 and 12, respectively [60].

### 4.3. Previous Theoretical Studies of HfO_2_

The use of amorphous hafnium oxides in semiconductors has led to a large theoretical effort to understand and improve these devices. Classical molecular dynamics simulations have provided an overview of the variety of structures over a wide temperature range. Large-scale MD simulations by Schie et al. found that the amorphous supercell exhibited pronounced first-nearest-neighbor peaks, small and broadened second-nearest-neighbor peaks, but no long-range order beyond 8 Å [2]. In addition, Hf is mainly found to form six- and seven-fold coordination polyhedra, with some five-, eight-, and nine-fold coordination polyhedra also evident, while for oxygen the ratio of three- to four-fold coordination changes from 1:1 in the monoclinic form to almost 2:1 in the amorphous form, along with the addition of two- and five-fold coordinated species [2]. This is accompanied by significant broadening of the peaks in the amorphous form.

Similar results for the local coordination have been obtained in density functional theory calculations. Cressoli et al. simulated amorphous HfO_2_ and also found a prevalence of six- and seven-fold coordinated Hf and three- and four-fold coordinated oxygen atoms [4]. However, there is a large variation in the predicted packing between the polyhedra in the different models. A study combining both classical MD and DFT calculations found domination of six-fold Hf atoms and three-fold O atoms led to significantly more corner-sharing polyhedral than edge sharing [61]. Other DFT calculations investigating the role of oxygen defects, namely the formation of oxygen vacancies and interstitial oxygens [5], have found lower average Hf–O and O–Hf coordination numbers (5.7 and 2.9, respectively). Another DFT study on O vacancy formation in amorphous HfO_2_ predicts that while the Hf–O bond length is close to that in m-HfO_2_, the O–O distribution is longer and broader than the crystalline phase [6].

Ab initio molecular dynamics simulations have recently been performed to generate an atomic structure model of amorphous hafnium oxide via the melt-and-quench scheme [3]. Upon melting, they found a significant increase in the number of three-coordinated O atoms and the appearance of some two-coordinated oxygen. Similar behavior was found in ab initio MD simulations by Nishio et al., who predicted a larger number of three coordinated and smaller number of four coordinated O atoms in the melt [7]. The amorphous structure generated by Scopel et al. was analyzed via bond-angle and partial pair distribution functions, yielding an average Hf–O nearest-neighbor distance of 2.2(3) Å, which is longer than the average value of 2.14 Å in the monoclinic crystal [3]. The most striking difference is the increase in width from 0.22 Å in the crystal to ~0.6 Å in the amorphous model. Scopel et al. note that this is broader than observed in the amorphous structures of ZrO_2_, and that experimental confirmation of this result is needed [3].

### 4.4. The Effect of Density on Amorphous HfO_2_ Structure

**Figure 7:**
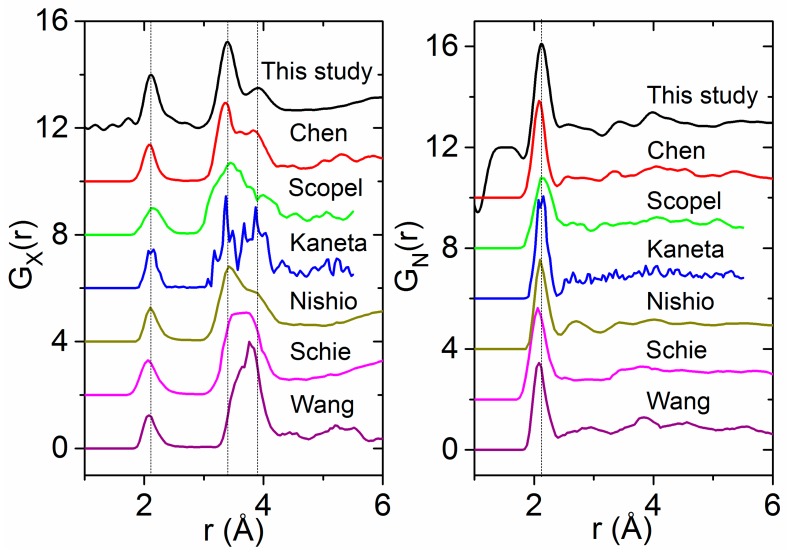
Comparison of our experimental pair distribution functions for amorphous HfO_2_ (black, top) with several molecular dynamics simulations taken from the literature [2,3,5,6,7,61] and weighted for: X-rays (**Left**); and neutrons (**Right**).

**Figure 8:**
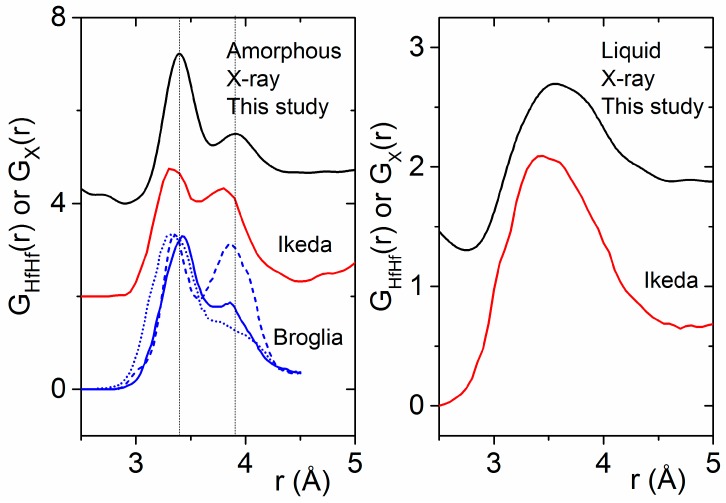
(**Left**) Comparison of the experimental X-ray pair distribution functions for amorphous HfO_2_ (black) with DFT simulations of the Hf–Hf partial pair distribution function taken from the literature [1,8]. Densities of 8.6, 10.19, and 11.5 g∙cm^−3^ are denoted by blue dashed, solid, and dotted lines respectively. (**Right**) The same comparison made for liquid HfO_2_.

Our experimentally-derived pair distribution functions for amorphous HfO_2_ are compared to X-ray- and neutron-weighted radial distribution functions from previous theoretical studies in Figure 7. A detailed comparison of the local atom–atom distances and coordination numbers measured in this study to the existing and current simulations is contained in Table 2. First principles MD simulations by Ikeda et al. using a melt slow quench method produce an amorphous phase similar in density to the liquid [8]. Two distinct Hf–Hf peaks are evident in the partial pair distribution functions obtained from simulations by Ikeda et al., Chen et al. and Broglia et al. [1,6,8] (Figure 7). Moreover, Broglia et al. demonstrated that higher densities lead to shorter bond lengths and O–O contact distances consistent with highly packed structures [1]. For monoclinic HfO_2_, the Hf–Hf partial is divided into two discrete peaks related to two distinct arrangements of the Hf-polyhedra. The region from 3.2 to 3.7 Å corresponds to ~30% edge-sharing (ES) polyhedra, and the longer distances between 3.8 and 4.2 Å correspond to ~70% (CS) corner sharing polyhedra. At low densities, these two peaks can be related to the relative distribution of CS and ES polyhedra in the amorphous form. At higher densities, the second peak broadens as ordering associated with a more open network breaks down, leading to a preferential ES polyhedral distribution (Figure 8).

Broglia et al. also showed that Hf–O–Hf angles are insensitive to density, and the Hf–O coordination number is most affected by density [1]. The average oxygen coordination number is primarily three-fold at low density and nearly four-fold at higher density. Similarly, the Hf–O coordination number increases with density from 6.1 to 7.6 (Table 2). However, our diffraction findings indicate that, even when the Hf–O coordination number in the melt and amorphous solid are similar, there can be substantial differences in polyhedral connectivity and intermediate range ordering. The distinct Hf–Hf correlations observed in the amorphous phase dissolve into a single broad peak in the melt (Figure 8).

It is well known experimentally that the structure of amorphous materials inherently depends on the method of synthesis and sample history. The same can be said for the theoretical framework used to predict the atomic structure. In low density models of HfO_2_, Ikeda et al. showed that NVT simulations leads to the formation of nanopores [8]. Similarly, Wang et al. chose a high enough density in their simulations to avoid holes in the network due to stresses appearing in the matrix [61]. We therefore have to consider how the amorphous samples used in this study are made. Gas phase condensation is known to produce fractal aggregates of nanoparticles [12]. The Q^−4^ rise at low-Q in the inset of Figure 1 indicates that the samples are an agglomeration of these smooth nanoparticles, and the low density of the material is associated with the packing of these particles rather than the generation of voids within the network. 

**Figure 9:**
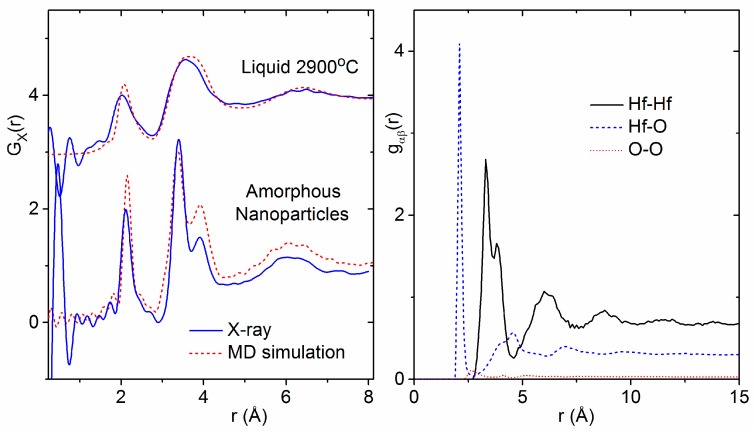
(**Left**) X-ray pair distribution functions of liquid HfO_2_ compared to the ab initio simulations performed in this study (shifted top, see text) and amorphous hafnia nanoparticles compared to the classical MD simulations performed in this study based on the parameters given by Broglia et al. [1]. (**Right**) The partial pair distribution functions for the amorphous nanoparticle simulation weighted by the Q = 0 factors for the X-ray case given in Equation (1).

**Figure 10:**
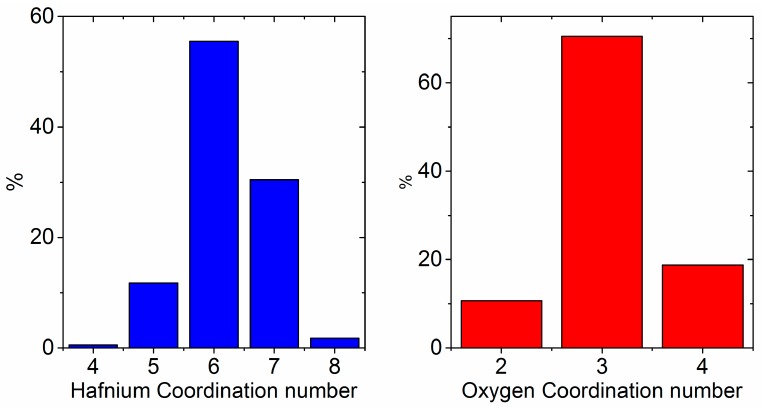
Probability distribution of coordination numbers corresponding to the local environments of Hf and O in amorphous HfO_2_ nanoparticles.

A crude agglomerate of 4 nanoclusters was made by tethering the mass of each sphere to prevent coalescing and minimizing the energy, then shrinking the cell until the clusters touch (Videos S2 and S3). We note that the experimental density of the amorphous phase, 7.69 g∙cm^−3^, is comparable to that of close-packed spheres of molten HfO_2_. The pair distribution function of the simulated amorphous nanoparticle agglomerate contains two Hf–Hf correlations at 3.4 Å and 3.9 Å, which is consistent with our experimental results (Figure 9). A coordination number analysis of the amorphous nanoparticle model yields somewhat lower average coordination numbers for Hf and O (6.2 and 3.1, respectively). The probability distribution for each polyhedral species is shown in Figure 10.

Ab initio MD simulations of the bulk liquid in the NPT ensemble were performed starting with the final geometry from a classical MD simulation as described in Broglia et al. [1] at the liquid density. The single broad Hf–Hf peak observed in the X-ray pair distribution functions was reproduced (Figure 9).

**Figure 11:**
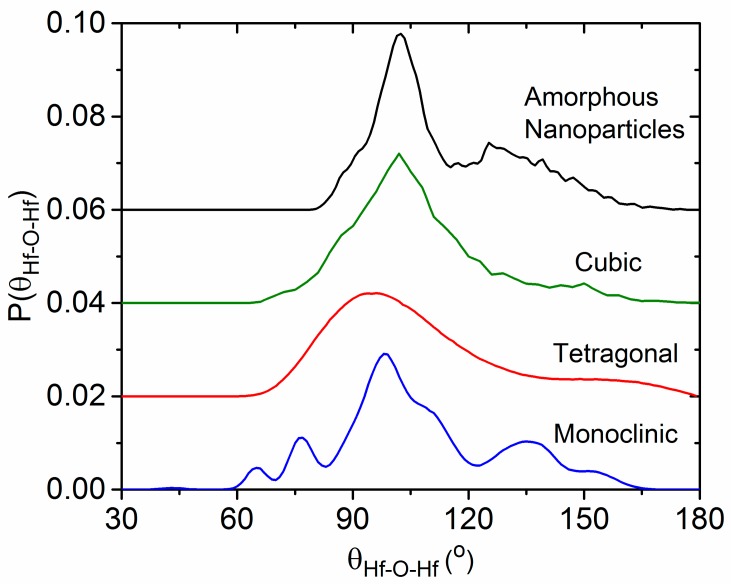
Distribution of bond angles monoclinic, tetragonal, and cubic HfO_2_ structures obtained from molecular dynamics simulations.

**Figure 12:**
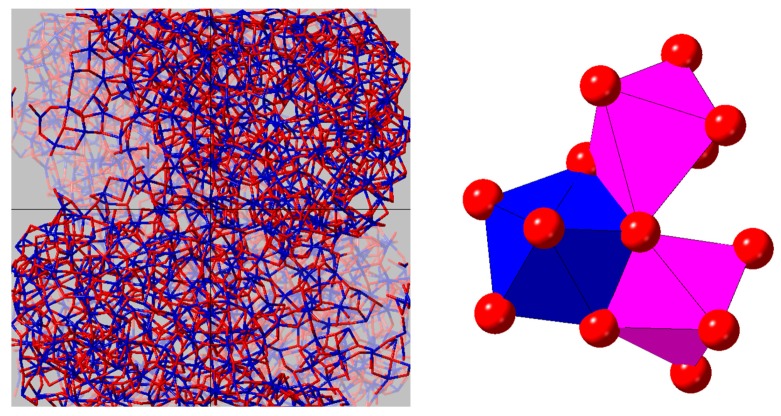
Snapshots taken from the MD model of amorphous hafnia nanoparticles: (**Left**) the agglomeration on nanoparticles within the simulation box; and (**Right**) edge- and corner-sharing HfO_n_ polyhedra in the low density amorphous form.

The Hf–O–Hf bond angle distribution analysis shown in Figure 11 shows a distinct difference between the distorted corner and edge shared HfO_7_ polyhedra present in the monoclinic phase compared to the solely edge shared HfO_8_ polyhedra that comprise the cubic phase. The principal peak at 102° in the cubic phase is in the same position as for the amorphous nanoparticles. The main difference is the addition of broad peak approximately centered around 135°, which is also observed in the monoclinic phase. We therefore attribute the 102° and 135° peaks to the existence of edge- and corner-sharing HfO_n_ polyhedra in our amorphous nanoparticles, as illustrated in Figure 12.

Here, we demonstrate that low density amorphous networks such as hafnia can show a combination of structural features observed in the liquid as well as the crystalline forms. Zhao et al. have predicted that ZrO_2_ “collapses into smaller clusters accompanied by spatial voids” at densities below 5.32 g/cm^3^ [63]. Cresoli and Vanderbilt assumed that HfO_2_ would behave the same at a similar atomic density, which corresponds to a density of 9.09 g/cm^3^ [4]. Concomitant with lower densities and void space, comes increased intermediate range ordering that is normally associated with a more open network, leading to the possibility of new families of disordered structures with varying properties. In this paper, diffraction methods are shown to be a stringent test for benchmarking the fundamental atom–atom interactions in a disordered material, and by comparing to the plethora of existing simulations on HfO_2_, significant populations of certain structures can be ruled out. Moreover, using experimental data to select realistic inter-atomic potentials, we present a methodology for predicting the likely topological networks associated amorphous nanoparticle structures based on their fabrication method.

## 5. Conclusions

A combination of complementary diffraction methods and MD simulations are used to characterize two very structurally different disordered forms of hafnia. The high-energy X-ray pair distribution functions are dominated by the Hf-correlations, and the neutron diffraction results are sensitive to the oxygen interactions and the presence of hydrogen contaminants. Experiments on the high temperature liquid at 2900 °C and an agglomeration of low-density amorphous nanoparticles made by gas condensation both reveal an average Hf–O coordination number of about seven, the same as that in the low temperature monoclinic phase. The main distinguishing feature in the atomic structure is the HfO_n_ polyhedral connectivity and topology. Edge sharing HfO_6,7_ polyhedra dominate the molecular dynamics simulations of the bulk liquid and amorphous nanoparticle clusters. The formation of distinct edge- and corner-sharing units leads to a splitting and sharpening of the Hf–Hf peak(s) in the amorphous pair distribution function, indicating a strong density dependence on structural connectivity of the network. Further investigation into bulk versus surface structure of the nanoparticle is in progress.

## Figures and Tables

**Table 1:** Dimensions and number of atoms in the simulation box for cubic, tetragonal, and monoclinic HfO_2_.

Phase	Number of Atoms	Box Dimensions
Cubic	1500	L*_x_* = L*_y_* = L*_z_* = 25.37
Tetragonal	2058	L*_x_* = L*_y_* = 25.04 and L*_z_* = 36.40
Monoclinic	1500	L*_x_* = L*_y_* = L*_z_* = 25.6419 *xy* = *yz* = 0.0 and *xz* = −4.47

**Table 2:** Comparison of local structure: Hf–O and Hf–Hf distances and coordination numbers for liquid and amorphous hafnia obtained from experiment and simulation. Coordination numbers, Hf–O bond lengths, and Hf–Hf distances for the cubic and monoclinic polymorphs are included for comparison.

Technique	Hf–O CN	Hf–O Peak (Å)	Hf–Hf Peaks (Å)	Density (g∙cm^−3^)	Reference
X/N-PDF a-HfO_2_	6.8(6)	2.13(1)	3.38(1), 3.89(1)	7.69	This study
X-PDF Liquid HfO_2_	7.0(6)	2.05(1)	3.67(2)	8.16	This study
Liquid ZrO_2_ *	5.9	2.1	3.7	4.98	Kohara et al. [62]
X-PDF on a-ZrO_2_*	6.7	2.1	-	-	Zhang et al. [60]
DFT	6.1	2.09(30)	3.0, 4.3	8.63	Chen et al. [6]
DFT	5.7	2.12	-	8.6	Kaneta et al. [5]
DFT	6.0	-	3.3, 3.8	-	Ikeda et al. [8]
DFT	6.44	-	-	9.39	Ceresoli et al. [4]
Ab initio MD	6.76	2.2(3)	3.45, 4.00	-	Scopel et al. [3]
Ab initio MD	6.57	2.08	3.5–3.6, 3.8	9.39	Nishio et al. [7]
Classical MD	6.13 6.92 7.63	2.112	3.35, 3.86 3.41, 3.86 3.32, 3.7–4.2	8.6 10.19 11.5	Broglia et al. [1]
MD	6.2	2.15	3.37, 3.92	7.69	This study
Classical MD	5.76	2.08	3.6	7.97	Wang et al. [61]
Classical MD	6.74	2.06	3.44, 3.72	7.85	Schie et al. [2]
Cubic	8	2.21	3.62	10.45 ^1^	Passerini et al. [31]
Monoclinic	7	2.13	3.43, 3.94	10.11	Hann et al. [57]

^1^ Correlations and density reported at room temperature. * ZrO_2_ data are included for comparison.

## Supplementary Materials

The following are available online at www.mdpi.com/1996-1944/10/11/1290/s1, Video S1: Simulation of individual amorphous HfO_2_ nanoparticle; Video S2: Melt and quench of lattice of a-HfO_2_ nanoparticles; Video S3: Contraction of a-HfO_2_ nanoparticle lattice.

## Appendix A

### Classical MD Simulations

The interactions between Hf and O in cubic, tetragonal and monoclinic phases of hafnium oxide were modeled using the modified charge transfer potential model (CTIP) developed by Zhou et al. [27,28]. This potential, which is an improved method upon Streitz–Mintmire potential [64], is made of two components: ionic and metallic. An electrostatic term is used to describe the ionic interactions in the oxide, (*E_es_*), while non-electrostatic interactions are captured by the embedded atom method, (*E_m_*). The total energy is as a sum of these two terms in Equation (A1).

*E_t_* = *E_es_* + *E_m_*,
(A1)

In the CTIP model, to prevent cations or anions from exceeding their valance charges, charge bounds are applied. In the modified CTIP, the electrostatic energy is given as below:

(A2) $$\begin{array}{c} E_{es}=E_{0}+\overset{N}{\underset{i=1}{\sum }}q_{i}X_{i}+\frac{1}{2}\overset{N}{\underset{i=1}{\sum }}\overset{N}{\underset{j=1}{\sum }}q_{i}q_{j}V_{ij}\\ +\overset{N}{\underset{i=1}{\sum }}\omega \left[1-\frac{q_{i}-q_{min,i}}{\left|q_{i}-q_{min,i}\right|}\right]{\left(q_{i}-q_{min,i}\right)}^{2}\\ +\;\overset{N}{\underset{i=1}{\sum }}\omega \left[1-\frac{q_{max,i}-q_{i}}{\left|q_{i}-q_{min,i}\right|}\right]{\left(q_{i}-q_{max,i}\right)}^{2}\; \end{array}$$

where *q_min,i_* and *q_max,i_* are the charge bounds of atom *i*, *q_min,i_* < *q_i_* < *q_max,i_*_._ The embedded atom method (EAM) is used to represent the non-electrostatic interactions in the metallic region as follows [27,64]:

(A3) $$E_{m}=\;\frac{1}{2}\overset{N}{\underset{i=1}{\sum }}\overset{i_{M}}{\underset{j=i_{1}}{\sum }}\phi_{ij}\left(r_{ij}\right)+\;\overset{N}{\underset{i=1}{\sum }}F_{i}\left(\rho_{i}\right)$$

In Equation (A3), *φ_ij_*(*r_ij_*) represents the pairwise interaction energy between atoms *i* and *j* that are at distance *r_ij_* apart. *N* is the total number of atoms, and *i_M_* is the number of neighbors for atom *i*. For an alloy system, the generalized elemental pair potential is given by [27]:

(A4) $$\Phi \left(r\right)=\;\frac{A\;\exp\left[\alpha \left(\frac{r}{r_{e}}-1\right)\right]}{1+{\left(\frac{r}{r_{e}}-\kappa \right)}^{20}}-\frac{B\exp\left[-\beta \left(\frac{r}{r_{e}}-1\right)\right]}{1+{\left(\frac{r}{r_{e}}-\lambda \right)}^{20}}$$

*F_i_* represents the embedding energy, i.e., energy required to embed an atom *i* into a local site with electron density *ρ_i_* [27]. In the above expression, *f_i_*(*r_ij_*) is the electron density at the site of atom *i* arising from atom *j* separated by distance *r_ij_*, which is given by [27]:

(A5) $$f\left(r\right)=\frac{f_{e}\;\exp\left[-\beta \left(\frac{r}{r_{e}}-1\right)\right]}{1+{\left(\frac{r}{r_{e}}-\lambda \right)}^{20}}$$

The embedding energy functions F are designed to work well over a wide electron density range. Sasikumar et al. in their work of titanium oxide used spline functions across different density ranges to obtain a smooth function [27].

The pair wise interaction of Hf and O (Hf–Hf, Hf–O and O–O) are described in the Equation (A4). In addition to the pair terms, the oxygen interactions also have an embedding energy based on the local electron density. Equation (A6) gives the functional form of the electron density:

(A6) $$f\left(r\right)=\frac{f_{e}\;\exp\left[-\gamma \left(\frac{r}{r_{e}}-1\right)\right]}{1+{\left(\frac{r}{r_{e}}-\nu \right)}^{20}}$$

The embedding function is defined as follows [27,28]:

(A7) $$F_{j}\left(\rho \right)=\overset{3}{\underset{i=0}{\sum }}F_{ij}{\left(\frac{\rho }{\rho_{e,j}}-1\right)}^{i};\;\rho_{min,j}\leq \rho \leq \rho_{max,j}$$

where *j* varies from 0 to *M* (the number of metal types in the alloy oxide; *M* = 1, here).
